# Efficacy and safety of perioperative use of non-steroidal anti-inflammatory drugs for preemptive analgesia in lumbar spine surgery: a systematic review and meta-analysis

**DOI:** 10.1186/s13741-023-00347-7

**Published:** 2023-11-23

**Authors:** Nanshan Ma, Ping Yi, Zhencheng Xiong, Haoning Ma, Mingsheng Tan, Xiangsheng Tang

**Affiliations:** 1 Shanghai University of Traditional Chinese Medicine, Shanghai, 201203 People’s Republic of China; 2 Department of Orthopaedic Surgery, China-Japan Friendship Hospital, Beijing, 100029 People’s Republic of China; 3 Department of Orthopaedic Surgery, West China Hospital, Chengdu, 610041 People’s Republic of China

**Keywords:** Preemptive analgesia, NSAIDs, Lumbar spine surgery

## Abstract

**Objective:**

Lumbar spine disorders have become an increasingly common health problem in recent years. Modern clinical studies have shown that perioperative analgesia at certain doses can reduce postoperative pain by inhibiting the process of peripheral sensitization and central sensitization, which is also known as “preemptive analgesia,” Non-steroidal anti-inflammatory drugs (NSAIDs) are a class of drugs that achieve antipyretic and analgesic effects by inhibiting cyclooxygenase (COX) and affecting the production of prostaglandins. Our meta-analysis aimed to assess the efficacy and safety of perioperative preemptive analgesia with non-steroidal anti-inflammatory drugs in patients with lumbar spine surgery.

**Methods:**

We searched PubMed, ScienceDirect, the Cochrane Library, and the Web of Science for randomized controlled trials (RCTs) that met the inclusion criteria. A total of 12 clinical studies were included to assess the efficacy and safety of perioperative NSAIDs preemptive analgesia for lumbar spine surgery.

**Result:**

Twelve studies, including 845 patients, met the inclusion criteria. The results showed that perioperative receipt of NSAIDs for preemptive analgesia was effective and safe. Patient’s postoperative morphine consumption (*P* < 0.05), visual analog scale (*P* < 0.05), and numerical rating scale (*P* < 0.05) were not statistically associated with postoperative complications (*P* > 0.05).

**Conclusion:**

Our findings suggest that NSAIDs are effective and safe for preemptive analgesia in the perioperative period of lumbar spine surgery and that more and better quality RCTs and more in-depth studies of pain mechanics are still needed.

**Supplementary Information:**

The online version contains supplementary material available at 10.1186/s13741-023-00347-7.

## Introduction

Lumbar spine disorders have become an increasingly common health problem in recent years. Modern research has shown that factors such as heavy lifting, genetic physiology, and prolonged physical activity can lead to high mechanical loads on the lumbar spine, which in turn can lead to lumbar spine disease (Jäger et al., 2013). While modern spine surgeons use different procedures to resolve lumbar axial back pain, reconstruct spinal stability, and reduce the risk of paralysis, a large number of postoperative pain cases have gradually attracted the attention of clinicians (Puvanesarajah et al., 2015). When peripheral nociceptors are activated by inflammatory mediators, pain signals are transmitted to the center via the superior conduction tracts of the spinal cord, and the center analyses the response and causes pain (Devin & McGirt, 2015).

KEHLET (Kehlet & Dahl, 1993) pioneered the concept of enhanced recovery after surgery (ERAS) in 1993, which refers to improving the patient’s postoperative recovery by reducing the pain that occurs during the perioperative period. The concept of preemptive analgesia was first introduced by Crile (Ji et al., 2018) in the early twentieth century and refers to the reduction of postoperative pain by administering analgesics before surgical incisions. Woolf, Wall, and others (Wall, 1994; Woolf & Chong, 1993; Woolf & Salter, 2000) later developed the concept based on Crile’s theory and suggested that analgesia could be achieved by inhibiting peripheral sensitization and central sensitization. Local tissue damage can lead to sensitization of peripheral nerve endings and central nerves, and low-intensity subthreshold stimulation can also cause pain sensation. Taking effective measures to relieve pain in advance and reduce the sensitivity of the central and peripheral nerves to pain before receiving harmful stimulation from surgery can accelerate the recovery of patients and significantly reduce the trauma of surgery on patients and postoperative pain compared to analgesia after pain occurs.

Non-steroidal anti-inflammatory drugs (NSAIDs) are a class of drugs with antipyretic, analgesic, and anti-inflammatory effects. In recent years, NSAIDs have been used in some surgical procedures for preemptive analgesia. To systematically investigate the role of non-steroidal anti-inflammatory drugs in preemptive analgesia in lumbar spine surgery and thus provide some support for the potential use of NSAIDs as preemptive analgesia in spinal surgery, we construct this meta-analysis.

## Methods and materials

### Search strategy

Two researchers independently searched multiple databases, including PubMed (1966 to January 1, 2022), ScienceDirect (1990 to January 1, 2022), the Cochrane CENTRAL (1966 to January 1, 2022), and Web of Science (1997 to January 1, 2022). When conducting relevant searches, MeSH terms were linked to the appropriate keywords using Boolean operators (AND or OR), including “non-steroidal anti-inflammatory drugs,” “preemptive analgesia,” and “lumbar spine surgery.” Two researchers read the titles, abstracts, and full text of the retrieved articles independently and, in turn, then screened them. If they had different opinions, a third researcher screened them again, and a group discussion was held in response to the disagreement. References of the selected articles were searched again to ensure that as many relevant studies as possible were included, and the results were discussed, examined, and synthesized. The PRISMA (Preferred Reporting Items for Systematic Reviews and Meta-analysis) statement was considered to be an important reference for this meta-analysis (Moher et al., 2009).

### Study strategy

This meta-analysis included only randomized controlled trials (RCTs) in which one arm used NSAIDs in the perioperative period and the other arm(s) used placebo or other controlled interventions. No previous history of spinal surgery in the included patients and one or more of the following indicators were used as the outcome indicators for the study: postoperative morphine consumption, visual analog scale, numerical rating scale, and adverse events. Articles that did not adequately report the full dataset or could not be extracted were excluded along with articles that were not available in full text and duplicate publications.

### Data extraction

Two researchers independently collected the data required, a third summarized the data, and the three discussed and resolved any disagreements. Basic data included the first author and year of publication, type of study, sample size (experimental: control), age (experimental: control), gender (experimental: control), body mass index (BMI) (experimental: control), type of medication used in the intervention, and outcome indicators. We have provided a narrative summary of data for both primary and secondary outcomes, using tables to summarize them. We extracted the outcome indicator data of perioperative NSAIDs with drug intervention and placebo or no intervention in the included literature and studied the heterogeneity between the NSAIDs group and the placebo group (such as placebo or no intervention). In this meta-analysis, postoperative morphine consumption was the primary outcome, morphine consumption was divided into different time points to measure the level of pain at different time points in the postoperative period, and our team has standardized the perioperative morphine consumption in all articles to mg. Visual analog scale (VAS,0 and 10 for no pain and worst pain, respectively), numerical rating scale (NRS, 0 and 10 for no pain and worst pain, respectively), and adverse effects were secondary observations (Bielewicz et al., 2022). And subgroup analysis of adverse reactions, effect sizes are expressed as risk ratio (RR). For articles where the full dataset was not available, we have sent an email to the corresponding author requesting the original data. For articles for which original data could not be provided, we have excluded the study based on exclusion criteria.

### Bias risk assessment

The Cochrane Handbook of Systematic Reviews was used to evaluate the quality of the RCTs screened in this meta-analysis (Higgins et al., 2011). Researchers assessed the risk of bias in each study using the Risk of Bias 1 (RoB1) tool and the quality of each included RCT with the risk of bias scale specifying: random sequence generation, allocation concealment, blinding of participants and personnel, blinding of outcome assessment, incomplete outcome data, selective reporting, and other bias. The researcher judged each factor as high risk of bias, low risk of bias, or unclear risk of bias by carefully reading the content of each study.

### Statistical analysis

This meta-analysis performed relevant subgroup analyses based on different outcome indicators and adverse events. When the included outcome indicators were continuous data, we used mean difference (MD) and 95% confidence intervals (CI) for analysis; when binary data were included, risk ratio (RR) and 95% CI were used. We used a random-effects model when *I*^2^ > 50; conversely, a fixed-effects model was used. The RevMan 5.4.1 and STATA 16.0 Windows software for statistical analysis of all data. We considered the results statistically supported when *P* < 0.05. Finally, we used sensitivity analysis to evaluate the stability of the results of the combined literature analysis.

## Results

### Search results

**Fig. 1:**
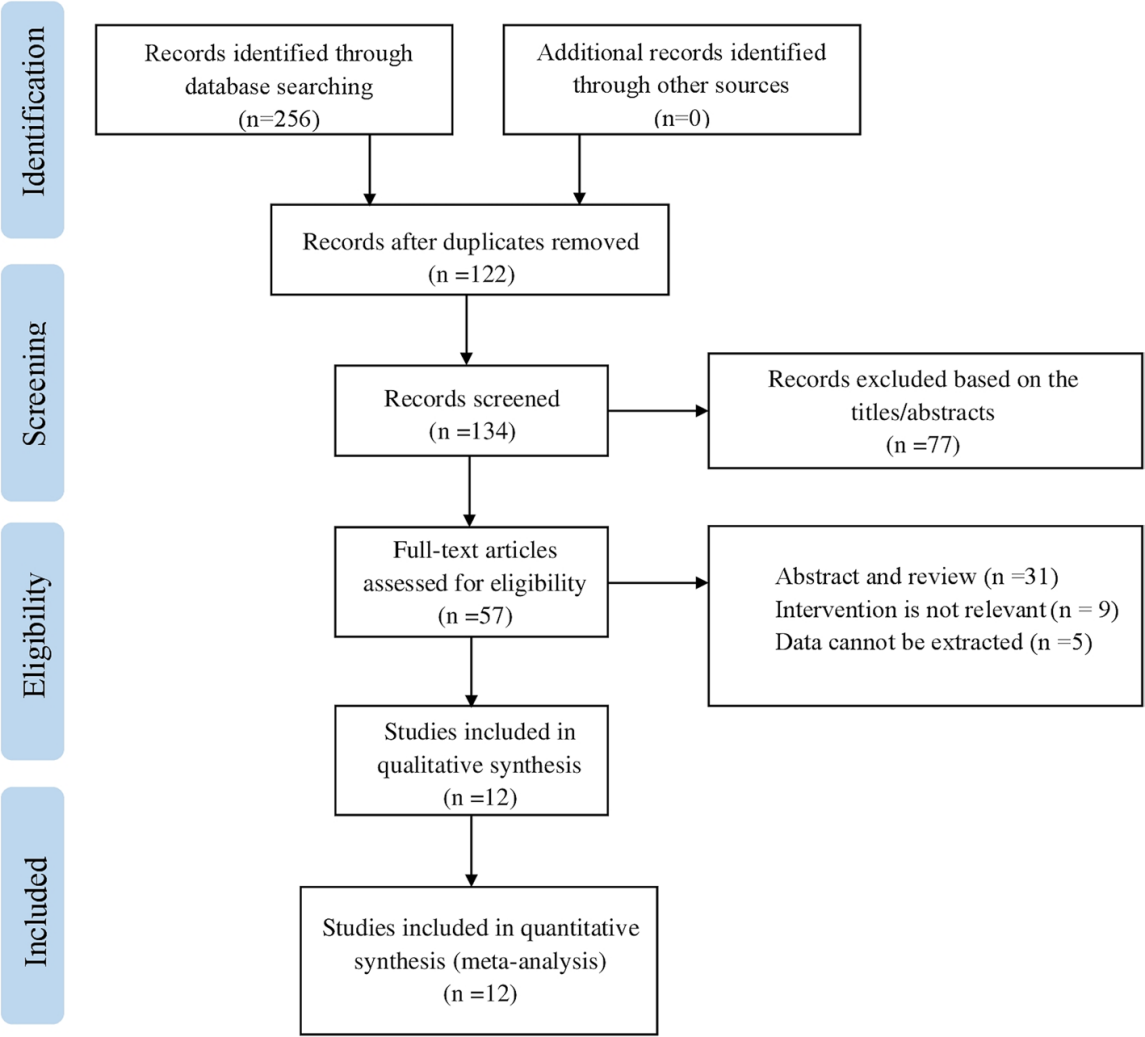
The search results for meta-analysis.

Based on the search strategy developed, we obtained an initial total of 256 studies. The two researchers each perused the titles, abstracts, and full texts of all articles retrieved, and a total of 57 articles passed the initial screening. These 57 studies were again evaluated by the two researchers according to the inclusion and exclusion criteria developed for this Meta-analysis. Finally,12 RCTs were included in the meta-analysis (Fig 1) (Cassinelli et al., 2008; Jirarattanaphochai et al., 2008; Karst et al., 2003; Kelsaka et al., 2014; Kien et al., 2019; Kim et al., 2016; Pookarnjanamorakot et al., 2002; Raja et al., 2019; Reuben et al., 1997; Riest et al., 2008; Rowe et al., 1992; Siribumrungwong et al., 2015).

### Study characteristics

In this meta-analysis, a total of 12 articles that met the inclusion criteria were included (Cassinelli et al., 2008; Jirarattanaphochai et al., 2008; Karst et al., 2003; Kelsaka et al., 2014; Kien et al., 2019; Kim et al., 2016; Pookarnjanamorakot et al., 2002; Raja et al., 2019; Reuben et al., 1997; Riest et al., 2008; Rowe et al., 1992; Siribumrungwong et al., 2015). A total of 12 studies were included and investigated the efficacy of NSAIDs in preemptive analgesia for lumbar spine surgery. A total of eight studies used postoperative morphine consumption as the primary outcome measure (Cassinelli et al., 2008; Jirarattanaphochai et al., 2008; Kien et al., 2019; Pookarnjanamorakot et al., 2002; Reuben et al., 1997; Riest et al., 2008; Rowe et al., 1992; Siribumrungwong et al., 2015), with each morphine consumption divided into four subgroups based on postoperative time. Eleven other studies assessed the difference in efficacy between the two groups of patients by pain intensity rating, including VAS and NRS (Cassinelli et al., 2008; Karst et al., 2003; Kelsaka et al., 2014; Kien et al., 2019; Kim et al., 2016; Pookarnjanamorakot et al., 2002; Raja et al., 2019; Reuben et al., 1997; Riest et al., 2008; Rowe et al., 1992; Siribumrungwong et al., 2015), with seven studies using VAS as an outcome indicator (Cassinelli et al., 2008; Karst et al., 2003; Kelsaka et al., 2014; Kien et al., 2019; Kim et al., 2016; Pookarnjanamorakot et al., 2002; Rowe et al., 1992) and four studies using NRS as an outcome indicator (Raja et al., 2019; Reuben et al., 1997; Riest et al., 2008; Siribumrungwong et al., 2015). Three studies reported adverse events including pruritus, nausea and vomiting, dyspepsia, and constipation (Jirarattanaphochai et al., 2008; Raja et al., 2019; Siribumrungwong et al., 2015). The recording of morphine consumption and pain scores (VAS, NRS) at different time points postoperatively can reflect the effect of NSAIDs used perioperatively on the pain relief of patients undergoing lumbar spine surgery at different time points (short or long-term) postoperatively. The characteristics of the studies included in the meta-analysis are listed in Table 1.

**Table 1:** Characteristics of all studies in the meta-analysis

Author	Study type	Sample size *N*: C	Mean age *N*: C	Gender M: F	Mean BMI *N*: C	Intervention	Outcome
Jirarattanaphochai et al.	Double-Blind RCT	120 (60:60)	51 ± 12.7: 52 ± 10.9	51: 69	23.6 ± 2.3: 23.9 ± 2.9	40 mg parecoxib 30 min before surgery (intravenous) 40 mg every 12 h for 48 h after surgery (intravenous)	1, 4
Karst et al.	Double-Blind RCT	20 (12:8)	NP	NP	NP	Celecoxib 200 mg twice a day for 72 h starting on the evening before surgery (oral)	2
Pookarnjanamorakot et al.	Double-Blind RCT	47 (27:20)	50.0 ± 13.1: 50.2 ± 12.2	15: 32	NP	40 mg piroxicam 1–3 h before surgery (oral) After surgery, 40 mg for 24 h, 20 mg for 48 h (oral)	1, 2
Riest et al.	Double-Blind RCT	160 (80:80)	NP	NP	NP	Parecoxib 40 mg twice a day throughout	1, 3
Siribumrungwong et al.	Double-Blind RCT	64 (32:32)	58.2 ± 9.5: 55.6 ± 14	22: 42	26.4 ± 3.2: 26 ± 4.8	30 mg ketorolac 30 min before surgery (intravenous)	1, 3, and 4
	Double-Blind RCT	64 (32:32)	58 ± 8.6: 55.6 ± 14	24: 40	26 ± 3.6: 26 ± 4.8	40 mg parecoxib 30 min before surgery (intravenous)	1, 3, and 4
Rowe et al.	Double-Blind RCT	30 (16:14)	NP	18: 12	NP	Indomethacin formulation 75 mg 2 h before surgery (oral)	1, 2
Kelsaka et al.	Double-Blind RCT	50 (25:25)	44.1 ± 10.7: 47.9 ± 10.9	20: 30	NP	Dexketoprofen 50 mg 10 min before surgery (intravenous)	2
Cassinelli et al.	Double-Blind RCT	25 (13:12)	62.3 ± 10: 65.9±10.1	NP	NP	15 mg/0.5 mL (age > 65) or 30 mg/0.5 mL (age < 65) at the time of the surgical wound closure, 6 h postoperative and 12 h postoperative.	1, 2
Kien et al.	RCT	60 (30:30)	44.93 ± 10.26: 48.23 ± 11.88	30: 30	NP	150 mg pregabalin, 200 mg of celecoxib 2 h before induction (oral)	1, 2
Kim et al.	RCT	80 (40:40)	67.9 ± 7.6: 66.3 ± 10	NP	NP	75 mg pregabalin, 500 mg acetaminophen, 10 mg extended-release oxycodone 1 h before surgery and twice daily after surgery 200 mg celecoxib before surgery and once daily after surgery	2
Raja et al.	Double-Blind RCT	97 (47:50)	49.7 ± 12.33: 51.6 ± 9.46	23: 74	26.4 ± 4.61: 25.8 ± 3.48	1 g paracetamol, 20 mg ketorolac, 75 mg pregabalin 4 h before surgery (oral)	3, 4
Scott et al.	Double-Blind RCT	40 (20:20)	46 ± 7: 41 ± 9	NP	NP	15 mg ketorolac every 6 h (intravenous)	1, 3
	Double-Blind RCT	40 (20:20)	45 ± 10: 41 ± 9	NP	NP	30 mg ketorolac every 6 h (intravenous)	1, 3

*N* NSAIDs group, *C* control group, *RCT* randomized controlled trial, *BMI* body mass index, *NP* not provide

Outcome: 1, morphine consumption; 2, Visual analog scale; 3, Numeric rating scale; 4, adverse event

### Risk of bias assessment

**Fig. 2:**
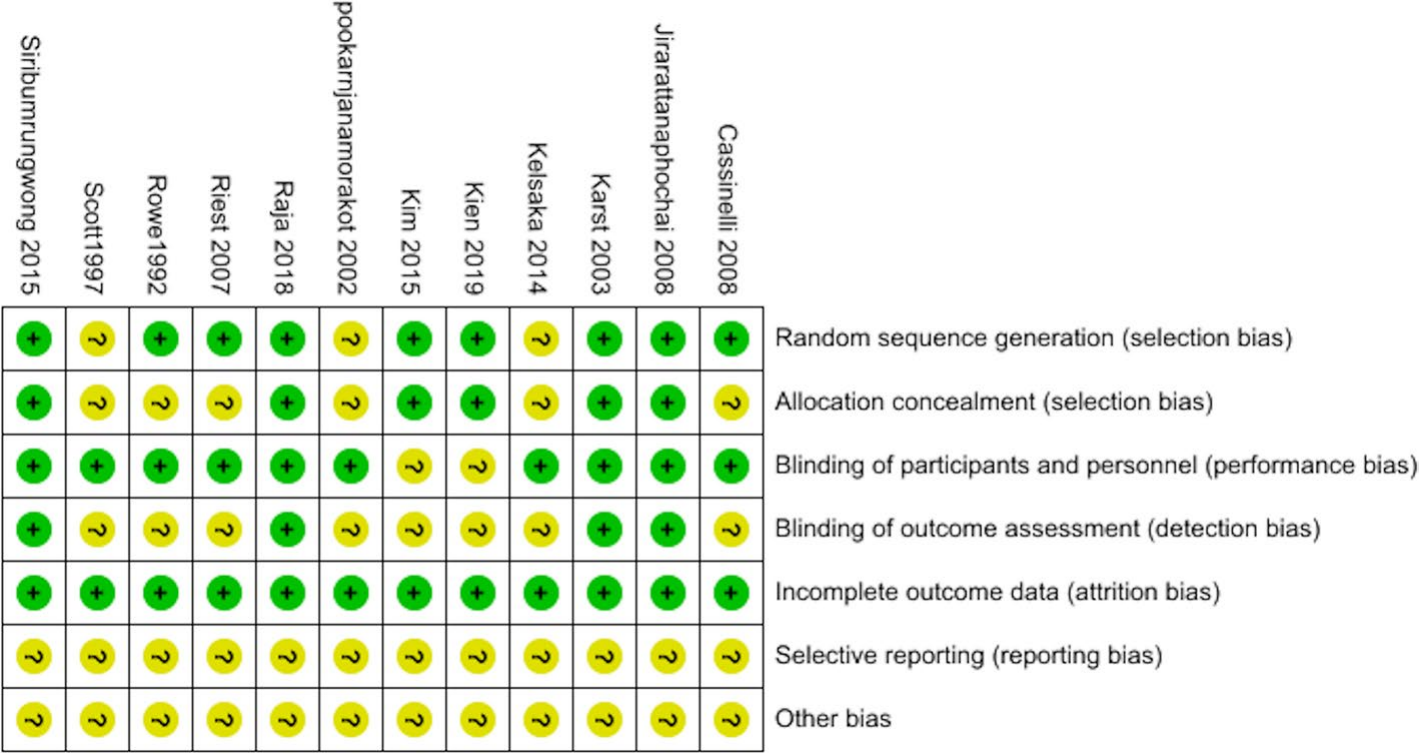
Risk of bias assessment graph.+, low risk; −, high risk; ?, unclear.

The risk of bias assessment for the included 12 RCTs is shown in Fig. 2 (Cassinelli et al., 2008; Jirarattanaphochai et al., 2008; Karst et al., 2003; Kelsaka et al., 2014; Kien et al., 2019; Kim et al., 2016; Pookarnjanamorakot et al., 2002; Raja et al., 2019; Reuben et al., 1997; Riest et al., 2008; Rowe et al., 1992; Siribumrungwong et al., 2015). Nine studies used random sequence generation and gave specific grouping methods (Cassinelli et al., 2008; Jirarattanaphochai et al., 2008; Karst et al., 2003; Kien et al., 2019; Kim et al., 2016; Raja et al., 2019; Riest et al., 2008; Rowe et al., 1992; Siribumrungwong et al., 2015), and six studies used allocation concealment and gave specific allocation schemes (Jirarattanaphochai et al., 2008; Karst et al., 2003; Kien et al., 2019; Kim et al., 2016; Raja et al., 2019; Siribumrungwong et al., 2015), ten studies were blinded (Cassinelli et al., 2008; Jirarattanaphochai et al., 2008; Karst et al., 2003; Kelsaka et al., 2014; Pookarnjanamorakot et al., 2002; Raja et al., 2019; Reuben et al., 1997; Riest et al., 2008; Rowe et al., 1992; Siribumrungwong et al., 2015), and in 12 studies, selective reporting and other biases could not be accurately determined (Cassinelli et al., 2008; Jirarattanaphochai et al., 2008; Karst et al., 2003; Kelsaka et al., 2014; Kien et al., 2019; Kim et al., 2016; Pookarnjanamorakot et al., 2002; Raja et al., 2019; Reuben et al., 1997; Riest et al., 2008; Rowe et al., 1992; Siribumrungwong et al., 2015). The quality of the included studies was acceptable.

### Results

#### Morphine consumption

**Fig. 3:**
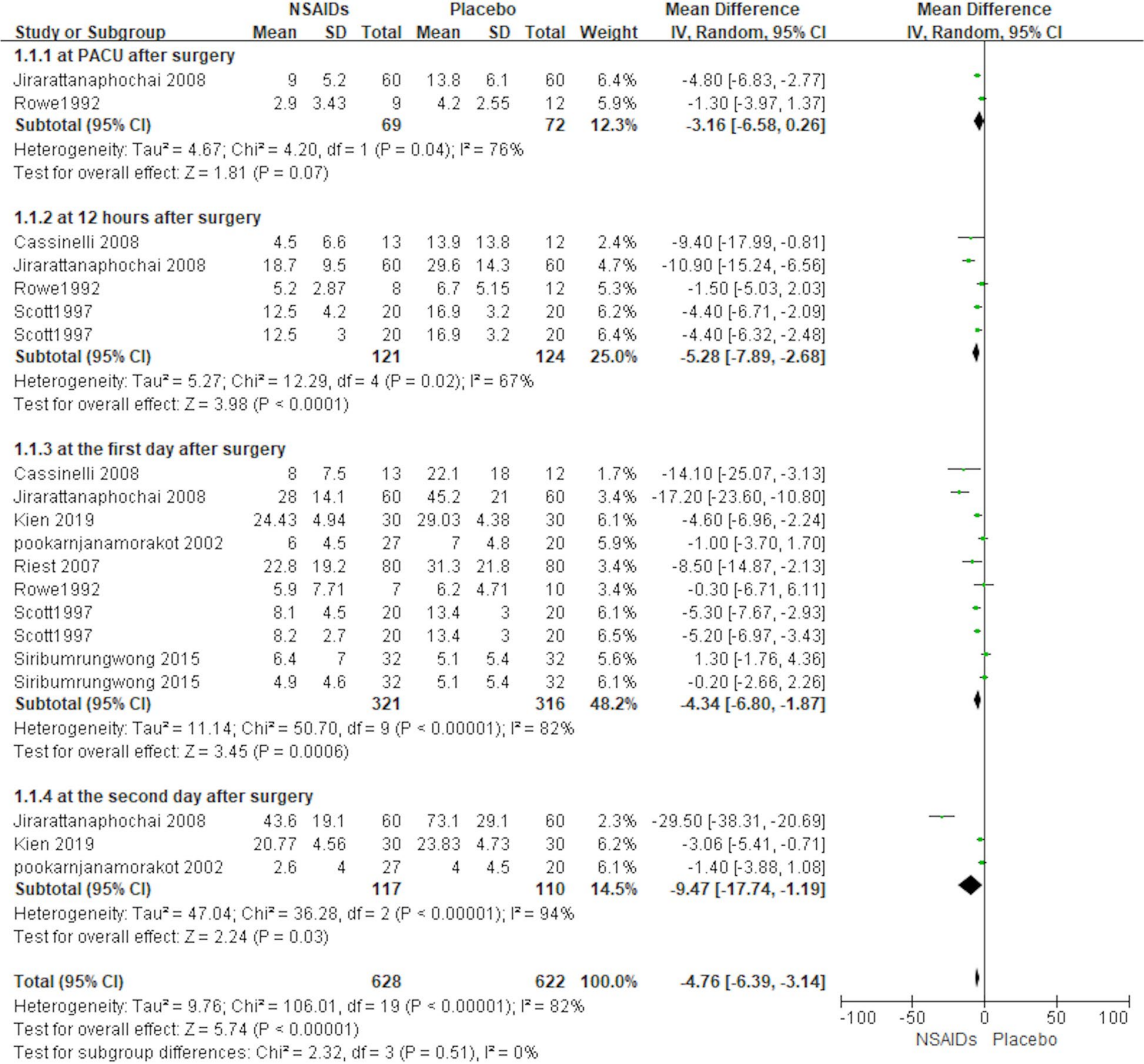
Forest plot showing the effect of the NSAIDs group and Placebo group on morphine consumption after lumbar spine surgery.

A total of 8 of the 12 included studies (598 patients) used postoperative morphine consumption as the primary outcome indicator (Cassinelli et al., 2008; Jirarattanaphochai et al., 2008; Kien et al., 2019; Pookarnjanamorakot et al., 2002; Reuben et al., 1997; Riest et al., 2008; Rowe et al., 1992; Siribumrungwong et al., 2015). There were four subgroups depending on different postoperative time points. The forest plot shows the effect of perioperative NSAID use on postoperative morphine consumption in the lumbar spine in Fig. 3. Two studies (Jirarattanaphochai et al., 2008; Rowe et al., 1992) provided data on postoperative PACU morphine consumption, 4 studies (Cassinelli et al., 2008; Jirarattanaphochai et al., 2008; Reuben et al., 1997; Rowe et al., 1992) provided data on 12 h postoperative morphine consumption, 8 studies (Cassinelli et al., 2008; Jirarattanaphochai et al., 2008; Kien et al., 2019; Pookarnjanamorakot et al., 2002; Reuben et al., 1997; Riest et al., 2008; Rowe et al., 1992; Siribumrungwong et al., 2015) provided data on 1 day postoperative morphine consumption, and 3 studies (Jirarattanaphochai et al., 2008; Kien et al., 2019; Pookarnjanamorakot et al., 2002) provided data on 2 days postoperative morphine consumption. As *I*^2^ was greater than 50%, a random effects model was used for this analytical procedure. There was a statistically significant difference in morphine consumption on 12 h postoperative, at 1 day postoperatively, and at 2 days postoperatively between the NSAIDs group and the placebo group (MD = −5.28, 95% CI (−7.89, −2.68), *P* < 0.05; MD = −4.34, 95% CI (−6.80, −1.87), *P* < 0.05; MD = −9.47, 95% CI (−17.74, −1.19), *P* < 0.05). There was not a statistically significant difference in morphine consumption in the postoperative PACU between the NSAIDs group and placebo group based on the results of the pooled analysis, [MD = −3.16, 95% CI (−6.58, 0.26), *P* > 0.05]. The results showed that patients who used NSAID drugs perioperatively consumed less morphine at 12 h, 1 day, and 2 days postoperatively than patients who used a placebo (*P* < 0.05).

#### VAS

**Fig. 4:**
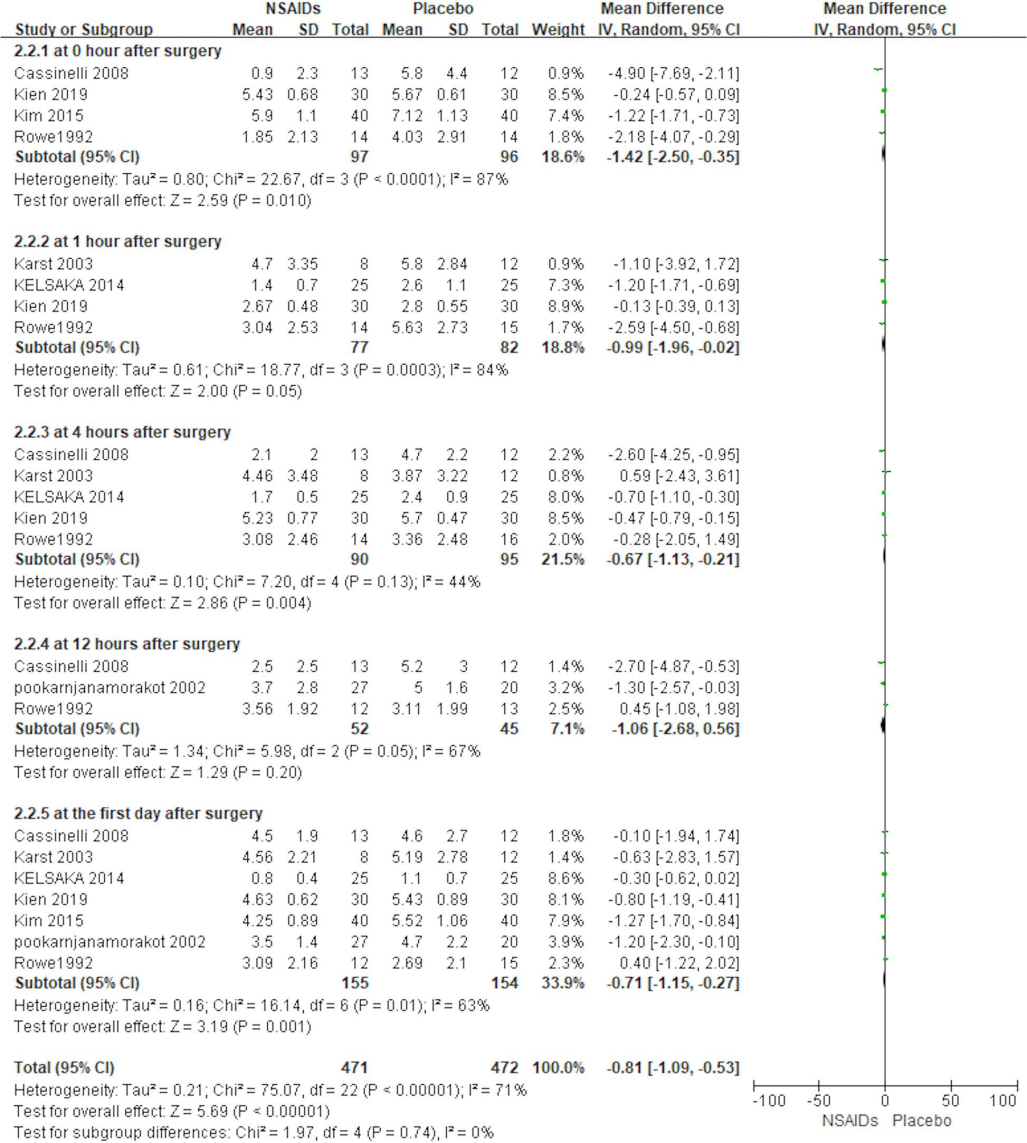
Forest plot showing the effect of the NSAIDs group and placebo group on VAS score after lumbar spine surgery.

A total of 7 of the 12 included studies (312 patients) used VAS as a secondary outcome measure (Cassinelli et al., 2008; Karst et al., 2003; Kelsaka et al., 2014; Kien et al., 2019; Kim et al., 2016; Pookarnjanamorakot et al., 2002; Rowe et al., 1992). There were five subgroups depending on different postoperative time points. The forest plot shows the effect of perioperative NSAIDs used in lumbar spine surgery on patients’ postoperative VAS in Fig. 4. As *I*^2^ was greater than 50%, we used a random effects model for the analysis. There was a statistically significant difference in VAS at 0 h, 1 h, 4 h, and at 1 day postoperatively between the NSAIDs group and the placebo group [MD = −1.42, 95% CI (−2.50, −0.35), *P* < 0.05; MD = −0.99, 95% CI (−1.96, −0.02), *P* = 0.05; MD = −0.67, 95% CI (−1.13, −0.21), *P* < 0.05; MD = −0.71, 95% CI (−1.15, −0.27), *P* < 0.05]. There was not a statistically significant difference in VAS at 12 h postoperatively between the NSAIDs group and placebo group based on the results of the pooled analysis, [MD = −1.06, 95% CI (−2.68, 0.56), *P* > 0.05]. The results showed that patients who used NSAID drugs perioperatively had smaller VAS scores at 0 h, 1 h, 4 h, and 1 day postoperatively than patients who used a placebo (*P* < 0.05).

#### NRS

**Fig. 5:**
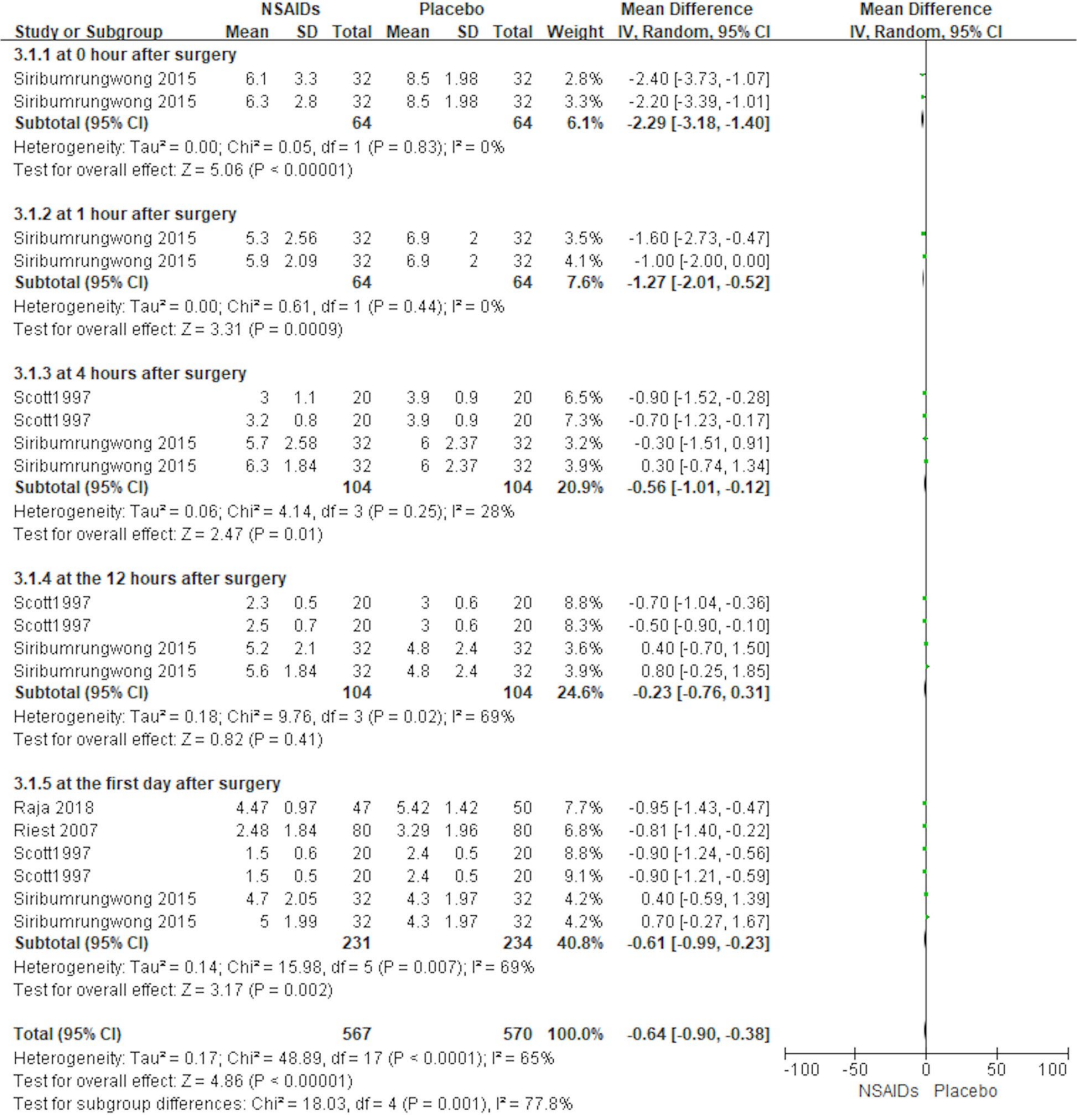
Forest plot showing the effect of the NSAIDs group and Placebo group on NRS score after lumbar spine surgery.

A total of 4 of the 12 included studies (413 patients) used NRS as a secondary outcome measure (Raja et al., 2019; Reuben et al., 1997; Riest et al., 2008; Siribumrungwong et al., 2015). There were five subgroups depending on different postoperative time points. The forest plot in Fig. 5 shows the effect of perioperative NSAIDs use on NRS in lumbar spine surgery. As *I*^2^ was greater than 50%, we used a random effects model for the analysis. There was a statistically significant difference in NRS at 0 h, 1 h, 4 h, and at 1 day postoperatively between the NSAIDs group and the placebo group [MD = −2.29, 95% CI (−3.18, −1.40), *P* < 0.05; MD = −1.27, 95% CI (−2.01, −0.52), *P* < 0.05; MD = −0.56, 95% CI (−1.01, −0.12), *P* < 0.05; MD = −0.61, 95% CI (−0.99, −0.23), *P* < 0.05]. There was not a statistically significant difference in VAS at 12 h postoperatively between the NSAIDs group and placebo group based on the results of the pooled analysis, [MD = −0.23, 95% CI (−0.76, 0.31), *P* > 0.05]. The results showed that patients who used NSAID drugs perioperatively had smaller NRS scores at 0 h, 1 h, 4 h, and 1 day postoperatively than patients who used a placebo (*P* < 0.05).

#### Adverse Events

**Fig. 6:**
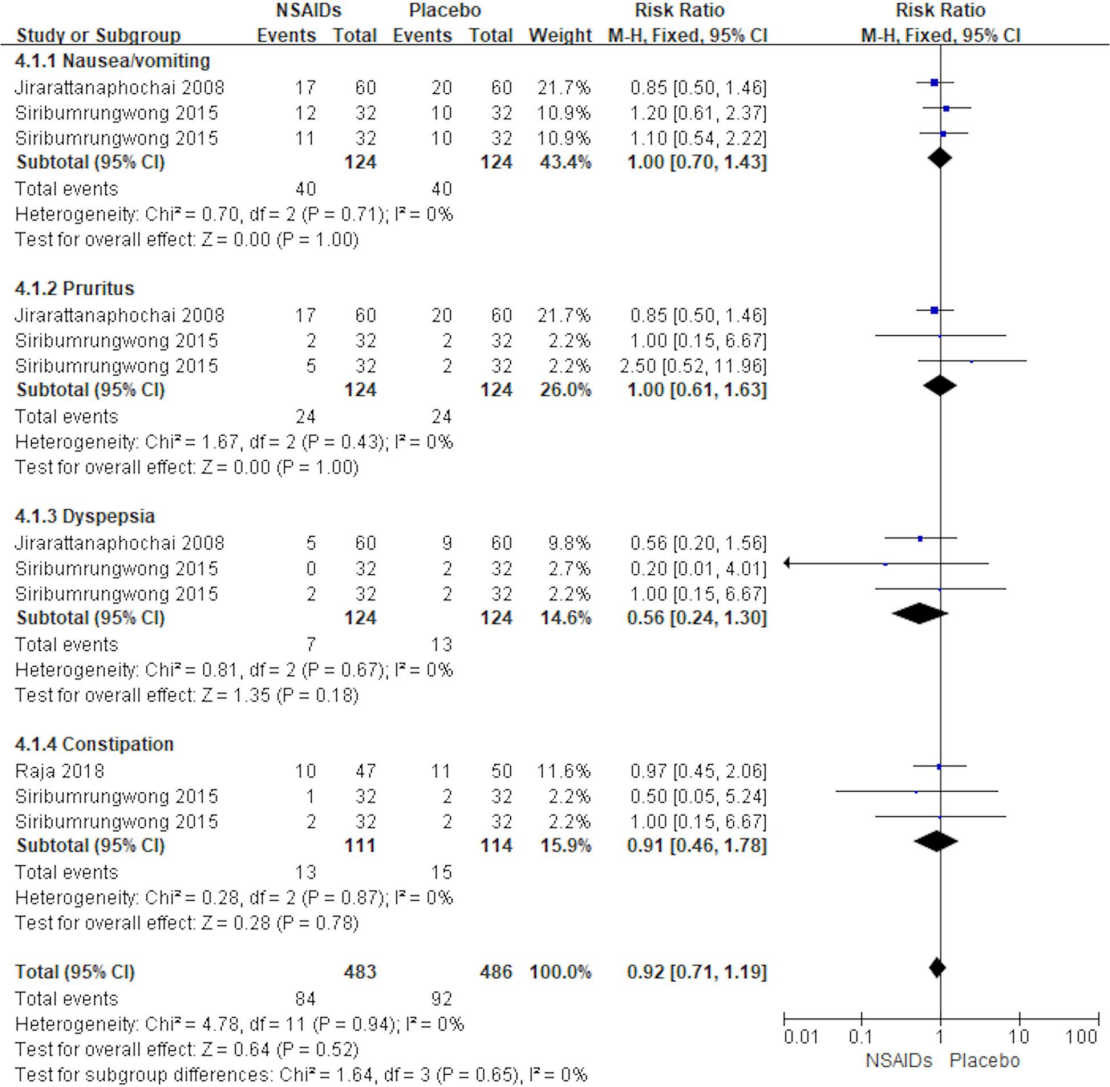
Forest plot showing the Effects of the NSAIDs group and placebo group on postoperative adverse events of lumbar spine surgery

Nausea and vomiting, dyspepsia, pruritus, and constipation were reported as adverse events in three articles (Jirarattanaphochai et al., 2008; Raja et al., 2019; Siribumrungwong et al., 2015). The forest plot shows the outcome of adverse events in the NSAIDs preemptive analgesia group versus the placebo group shown in Fig. 6. As *I*^2^ was less than 50%, we used a fixed effects model for the analysis. There was not a statistically significant difference in adverse event rates postoperatively between the NSAIDs group and placebo group based on the results of the pooled analysis, [nausea/vomiting, MD = 1.00, 95% CI (0.70, 1.43), *P* = 1.00; pruritus, MD = 1.00, 95% CI (0.61, 1.63), *P* = 1.00; dyspepsia, MD = 0.56, 95% CI (0.24, 1.30), *P* = 0.18; constipation, MD = 0.91, 95% CI (0.46, 1.78), *P* = 0.78]. The results showed that there were no statistically significant postoperative adverse effects in patients who used NSAID drugs in the perioperative period compared to those who used a placebo.

### Publication bias

**Fig. 7:**
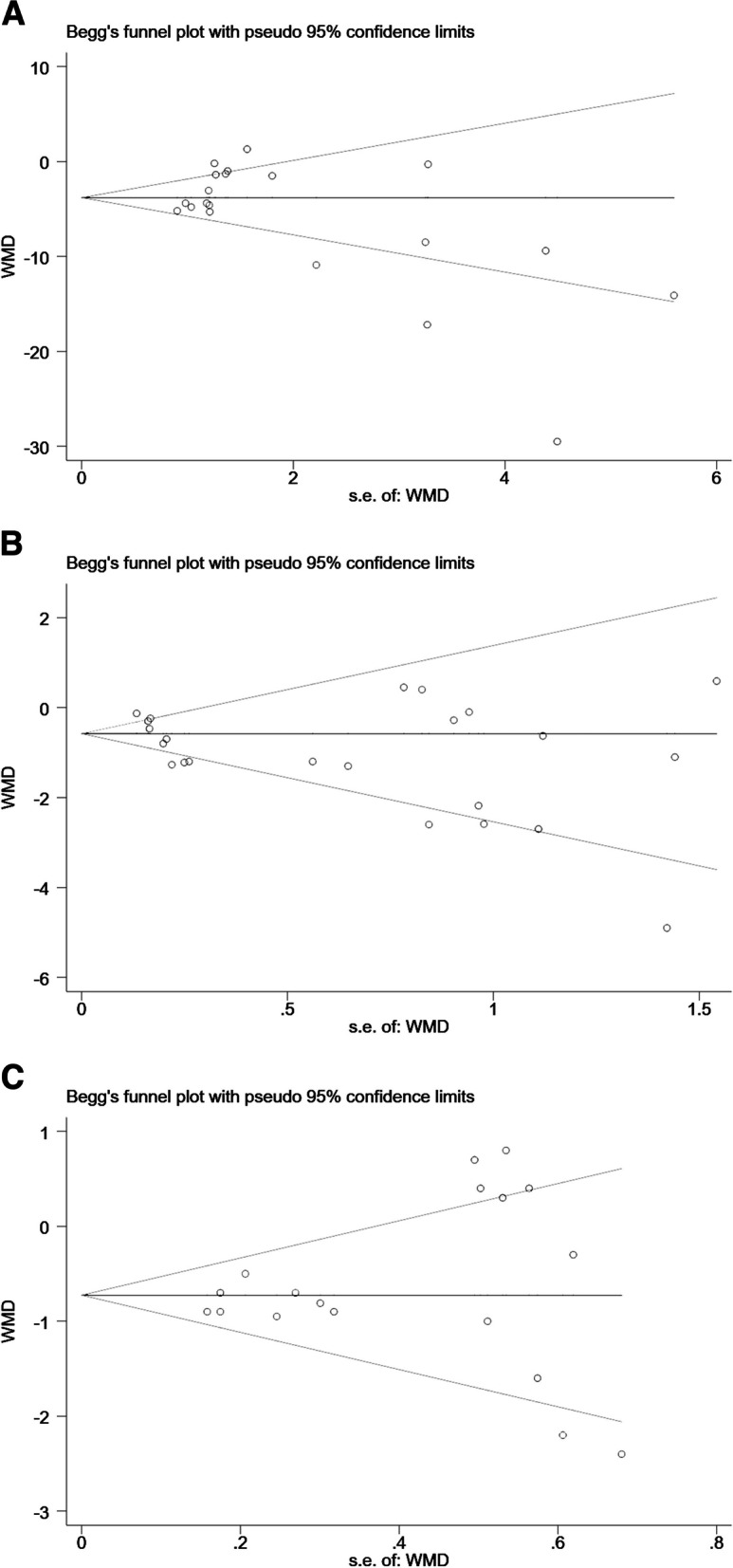
Begg’s funnel plot in this meta-analysis. **a** Morphine consumption. **b** VAS. **c** NRS

**Fig. 8:**
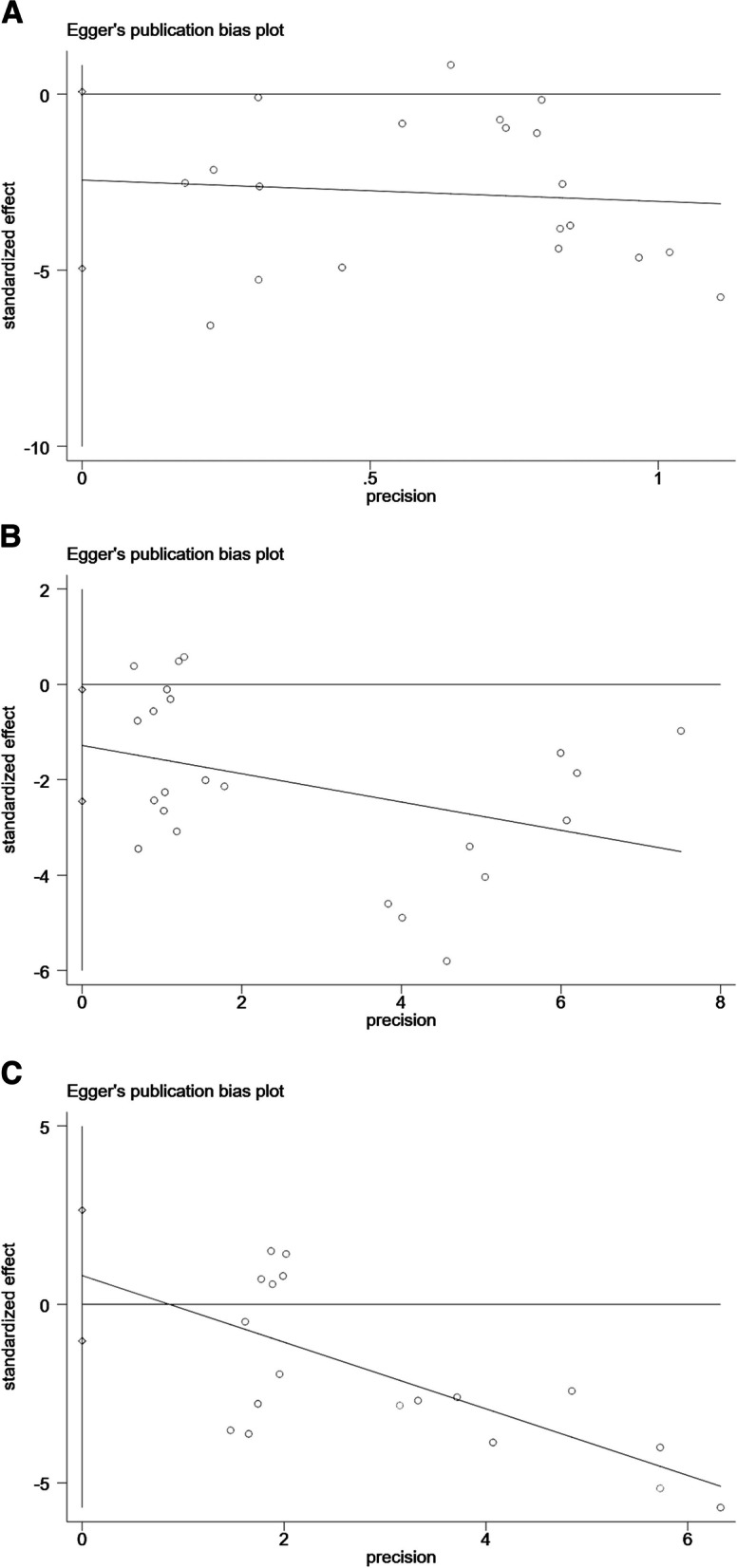
Egger’s funnel plot in this meta-analysis. **a** Morphine consumption. **b** VAS. **c** NRS

The high heterogeneity of results for some of the subgroups analyzed in this meta-analysis was considered to be related to differences in the type of drug used, drug dose, method of drug administration, frequency of drug administration in each of the included studies, and the quality and number of articles included. Therefore, for outcome indicators with high heterogeneity in this meta-analysis, we used Begg’s test and Egger’s test to assess publication bias. The results showed that *P* < 0.05 for Egger’s test for VAS, suggesting a possible publication bias for VAS. The other outcome indicators Begg’s test and Egger’s test were both *P* > 0.05, indicating no publication bias for any other outcome indicator[morphine consumption subgroup, Begg’s test *P* = 0.347, Egger’s test *P* = 0.056. VAS subgroup, Begg’s test *P* = 0.245, Egger’s test *P* = 0.034. NRS subgroup, Begg’s test *P* = 1, Egger’s test *P* = 0.364], shown in Figs. 7 and 8.

### Sensitivity analysis

**Fig. 9:**
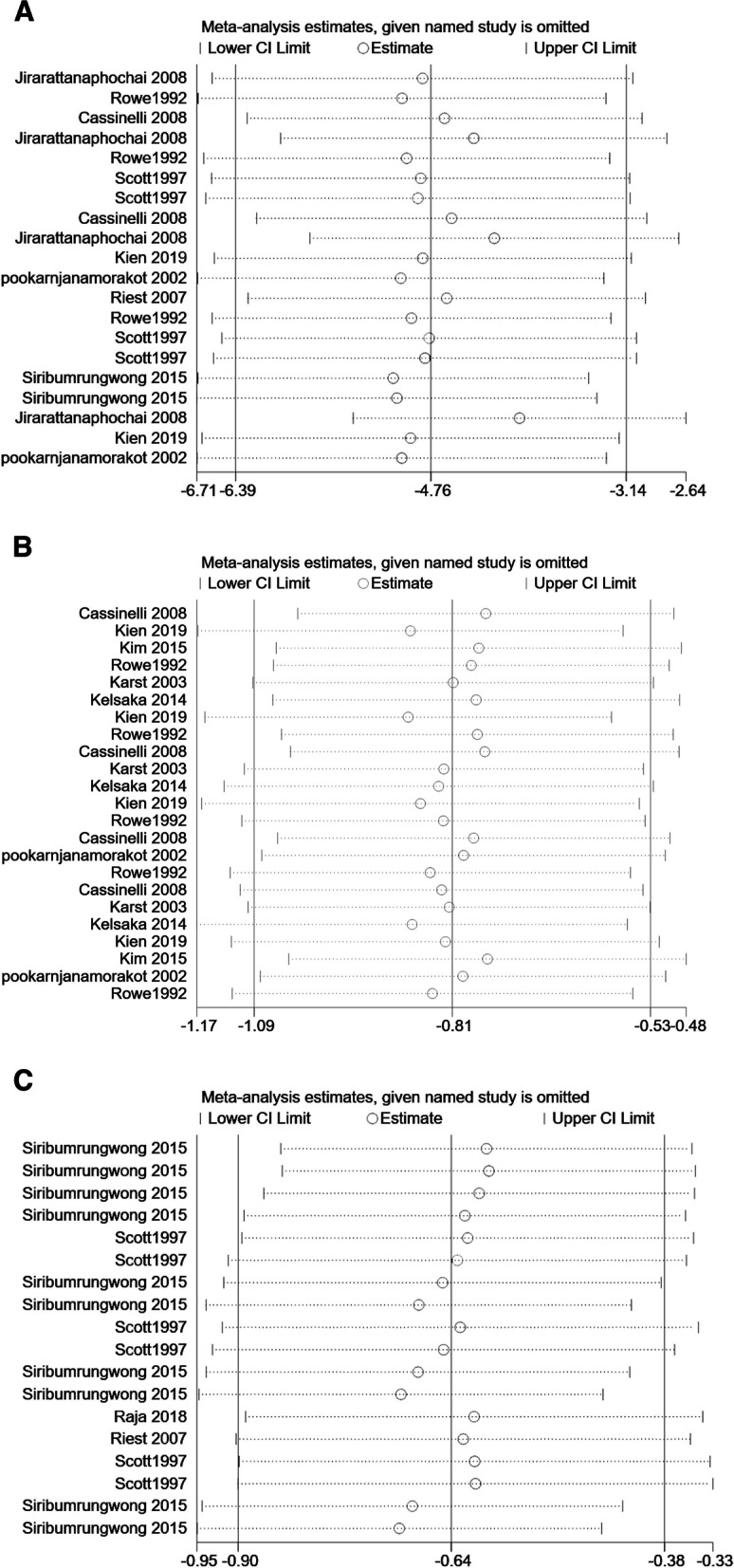
Sensitivity analysis in this meta-analysis. **a** Morphine consumption. **b** VAS. **c** NRS

We performed sensitivity analyses on outcome indicators that showed a high degree of heterogeneity. It showed no significant change when we excluded all outcome indicators from the included 12 studies one by one and found, indicating that the sensitivity analysis was robust, as shown in Fig. 9.

## Discussion

This meta-analysis examines the use of NSAIDs for preemptive analgesia in lumbar spine surgery. The traditional posterior lumbar incision is large, especially when multi-segmental lumbar lesions are encountered, requiring a larger field to be exposed, longer incisions, more internal fixation implants, and longer operative times, as well as causing more damage to the patient and more severe postoperative pain, which not only does it affect the patient’s early postoperative recovery, but some patients may also develop long-term pain syndrome after surgery, which subsequently affects the efficacy of the procedure (Small & Laycock, 2020). Costelloe (Costelloe et al., 2020) analyzed a total of 21 studies published from 2000 to 2019 related to postoperative persistent pain (PPP) in patients undergoing spinal surgery. Many studies have shown that the development of chronic pain after spinal surgery is associated with the use of preoperative analgesic drugs. Although traditional analgesic drugs, opioids, are more potent and long-lasting compared to NSAIDs, they are prone to tolerance and dependence with long-term use, drug flooding and withdrawal, and even nociceptive sensitization reactions that manifest as increased pain, making it more difficult for clinicians to effectively control patients’ pain (Roozekrans et al., 2018), making it crucial to find effective and safe analgesic methods. NSAIDs exert their anti-inflammatory and analgesic effects by blocking the production of prostaglandins through inhibition of the cyclooxygenase (COX) isoenzyme. COX-1 enzymes are ubiquitous throughout the body, while COX-2 enzymes are more specific to acute and chronic inflammatory tissues. In spinal surgery, non-steroidal anti-inflammatory drugs are recognized as non-opioids (Rivkin & Rivkin, 2014; Kurd et al., 2017; Sinatra, 2002; Smith et al., 2019; Szatmári et al., 2019). Today, NSAIDs have been widely used for preoperative analgesia in a variety of major surgeries such as colorectal surgery and radical cystectomy, and are receiving increasing attention from clinicians in spine surgery (Rowe et al., 1992; Siribumrungwong et al., 2015; Small & Laycock, 2020). Several studies have reported stronger postoperative analgesia with NSAIDs perioperatively than with a placebo alone. So we compiled relevant RCTs and constructed this meta-analysis to evaluate the effectiveness and safety of perioperative NSAID preceding analgesia in lumbar spine surgery.

In this meta-analysis, a total of 12 articles that met the inclusion criteria were included (Cassinelli et al., 2008; Jirarattanaphochai et al., 2008; Karst et al., 2003; Kelsaka et al., 2014; Kien et al., 2019; Kim et al., 2016; Pookarnjanamorakot et al., 2002; Raja et al., 2019; Reuben et al., 1997; Riest et al., 2008; Rowe et al., 1992; Siribumrungwong et al., 2015). NSAIDs were used in all studies’ experimental groups for preemptive analgesia in the perioperative period. Meanwhile, the control group used a placebo, and all patients in this meta-analysis were undergoing lumbar spine surgery. In the study indicator of postoperative morphine consumption, we classified it into four types according to the time of measurement, including at the time of postoperative PACU, 12 h, 1 day, and 2 days after surgery. By analyzing the results of the above study, it was concluded that at 12 h, 1 day, and 2 days postoperatively, postoperative morphine use was lower in patients with perioperative NSAIDs preemptive analgesia than in the placebo group. The pooled analysis of postoperative VAS showed that at 0 h postoperatively, 1 h, 4 h postoperatively, and 1 day postoperatively patients with perioperative NSAIDs preemptive analgesia had lower VAS scores than the placebo group at all four time points. The NRS results showed that postoperatively, at 0 h postoperatively, 1 h, 4 h postoperatively, and 1 day postoperatively patients with perioperative NSAIDs preemptive analgesia had lower NRS scores than the placebo group at all four time points. Pooled analysis of postoperative complications showed no significant difference in the incidence of postoperative complications between the NSAIDs analgesia and placebo groups.

Our results showed that for patients receiving NSAIDs during the perioperative period, VAS scores and NRS scores at 0 h, 1 h, 4 h, and 1 day postoperatively, morphine consumption at 12 h, 1 day, and 2 days postoperatively were significantly lower than those in the placebo group. This suggests that NSAIDs can significantly reduce postoperative pain and morphine consumption in patients in the short term. In addition, there is no statistically significant postoperative adverse reaction between the NSAIDs group and the placebo group. In summary, NSAIDs are effective and safe for preemptive analgesia in the perioperative period of lumbar spine surgery. The use of NSAIDs in the perioperative period of lumbar spine surgery can significantly reduce postoperative pain, which is worthy of clinical promotion.

### Limitations

There are limitations to this meta-analysis due to the small amount of included studies and the low quality. Firstly, the number of RCTs in this study was limited and many studies lacked elements such as blinding, allocation concealment, and selective reporting, resulting in poor-quality studies. Secondly, some of the outcome indicators showed high heterogeneity in this meta-analysis. Finally, some of the studies do not provide specific surgical protocols and this meta-analysis does not allow for further subgroup analysis of the different types of surgery.

## Conclusion

This meta-analysis aimed to evaluate the use of NSAIDs for preemptive analgesia in lumbar spine surgery. The results of this analysis showed that perioperative use of NSAIDs for preemptive analgesia provided more significant pain relief, lower postoperative morphine consumption, and pain scores compared to the placebo group, and that postoperative complication did not correlate with the use of NSAIDs. More and better quality randomized controlled trials (RCTs) and more in-depth studies of pain mechanics are still needed.

### Supplementary Information

**Additional file 1:.** Search strategy

## Data Availability

The data supporting this meta-analysis is from previously reported studies and datasets, which have been cited.
