# Supplementary material for: Efficacy and safety of perioperative use of non-steroidal anti-inflammatory drugs for preemptive analgesia in lumbar spine surgery: a systematic review and meta-analysis
Source: Perioper Med (Lond). 2023 Nov 23;12:61. doi: 10.1186/s13741-023-00347-7 (PMC10668431; doi:10.1186/s13741-023-00347-7)
Supplement: Supplementary file 1 — Additional file 1:. Search strategy [file 13741_2023_347_MOESM1_ESM.docx]

Search strategy:

When conducting relevant searches, MeSH terms were linked to the appropriate keywords using Boolean operators (AND or OR), including “non-steroidal anti-inflammatory drugs”, “preemptive analgesia”, and “lumbar spine surgery”.

Pubmed:

("lumbarised"[All Fields] OR "lumbarization"[All Fields] OR "lumbarized"[All Fields] OR "lumbars"[All Fields] OR "lumbosacral region"[MeSH Terms] OR ("lumbosacral"[All Fields] AND "region"[All Fields]) OR "lumbosacral region"[All Fields] OR "lumbar"[All Fields]) AND ("surgery"[MeSH Subheading] OR "surgery"[All Fields] OR "surgical procedures, operative"[MeSH Terms] OR ("surgical"[All Fields] AND "procedures"[All Fields] AND "operative"[All Fields]) OR "operative surgical procedures"[All Fields] OR "general surgery"[MeSH Terms] OR ("general"[All Fields] AND "surgery"[All Fields]) OR "general surgery"[All Fields] OR "surgery s"[All Fields] OR "surgerys"[All Fields] OR "surgeries"[All Fields]) AND ("anti inflammatory agents non steroidal"[Pharmacological Action] OR "anti inflammatory agents, non steroidal"[MeSH Terms] OR ("anti inflammatory"[All Fields] AND "agents"[All Fields] AND "non steroidal"[All Fields]) OR "non-steroidal anti-inflammatory agents"[All Fields] OR ("nonsteroidal"[All Fields] AND "anti"[All Fields] AND "inflammatory"[All Fields] AND "drugs"[All Fields]) OR "nonsteroidal anti inflammatory drugs"[All Fields]) AND ("analgesia"[MeSH Terms] OR "analgesia"[All Fields] OR "analgesias"[All Fields])
